# PEPR: pipelines for evaluating prokaryotic references

**DOI:** 10.1007/s00216-015-9299-5

**Published:** 2016-03-02

**Authors:** Nathan D. Olson, Justin M. Zook, Daniel V. Samarov, Scott A. Jackson, Marc L. Salit

**Affiliations:** Biosystems and Biomaterials Division, Material Measurement Laboratory, National Institute of Standards and Technology, Gaithersburg, MD USA; Statistical Engineering Division, Information Technology Laboratory, National Institute of Standards and Technology, Gaithersburg, MD USA; Department of Bioengineering, Stanford University, Stanford, CA USA

**Keywords:** Microbiology, Whole genome sequencing, Bioinformatics

## Abstract

The rapid adoption of microbial whole genome sequencing in public health, clinical testing, and forensic laboratories requires the use of validated measurement processes. Well-characterized, homogeneous, and stable microbial genomic reference materials can be used to evaluate measurement processes, improving confidence in microbial whole genome sequencing results. We have developed a reproducible and transparent bioinformatics tool, PEPR, Pipelines for Evaluating Prokaryotic References, for characterizing the reference genome of prokaryotic genomic materials. PEPR evaluates the quality, purity, and homogeneity of the reference material genome, and purity of the genomic material. The quality of the genome is evaluated using high coverage paired-end sequence data; coverage, paired-end read size and direction, as well as soft-clipping rates, are used to identify mis-assemblies. The homogeneity and purity of the material relative to the reference genome are characterized by comparing base calls from replicate datasets generated using multiple sequencing technologies. Genomic purity of the material is assessed by checking for DNA contaminants. We demonstrate the tool and its output using sequencing data while developing a *Staphylococcus aureus* candidate genomic reference material. PEPR is open source and available at https://github.com/usnistgov/pepr.

**Electronic supplementary material:**

The online version of this article (doi:10.1007/s00216-015-9299-5) contains supplementary material, which is available to authorized users.

## Introduction

Over the past decade, the availability of affordable and rapid Next-Generation Sequencing (NGS) technology has revolutionized the field of microbiology. Arguably the most discriminatory typing method available, whole genome sequencing (WGS), has been adopted by the research community, as well as public health laboratories, clinical testing laboratories, and the forensic community. High stakes decisions are often made based on the outcome of a WGS assay. To increase confidence in WGS assay, results a critical assessment of the errors inherent to the measurement processes is required. A number of sources of error associated with the WGS measurement process have been identified, but the degree to which they can be predicted, controlled, or compensated varies significantly [1].

Well-characterized, homogeneous, and stable genomic materials can be used to evaluate methods and aid in establishing confidence in results from a measurement process. For example, we recently characterized a whole human genome reference material (National Institute of Standards and Technology, NIST, Reference Material 8398) to assess the performance of variant calling in human genomic samples [2], but no reference materials for microbial sequencing currently exist. NIST is developing four microbial genomic DNA candidate reference materials to meet this need. When considering the extensive genomic diversity of prokaryotic organisms as well as the rapidly evolving and diverse DNA sequencing applications, we envision the need for a wide variety of application-specific genomic materials for use in method validation and benchmarking. Currently, many laboratories and sequencing centers are using in-house materials as part of a regular method validation and quality control system. However, the degree to which these materials are characterized varies significantly, and, therefore, a common pipeline for characterizing prokaryotic genomic materials is needed.

PEPR, Pipelines for Evaluating Prokaryotic References, a set of reproducible and transparent bioinformatic pipelines, was developed to characterize genomic materials for use in WGS method validation. Using the pipeline increases confidence in method validation through the ability to develop better-characterized control materials. PEPR characterizes prokaryotic genomic material for purity and homogeneity of the genome sequence, as well as the presence of genomic material other than the material genus. The general approach to material characterization that guided the development of PEPR is the use of orthogonal sequencing methods along with technical replicates to obtain consensus values for the characterized properties. These consensus values are our best current estimates of the true values. We do not assert probabilistic estimates of confidence or confidence classification values with the sequence data, as we lack good models of biases or systematic errors of current sequencing technologies. Here we will first describe PEPR then show how PEPR was used to characterize NIST *Staphylococcus aureus* genomic DNA candidate reference material.

## Methods

### Pipelines for evaluating prokaryotic references: PEPR

**Fig. 1:**
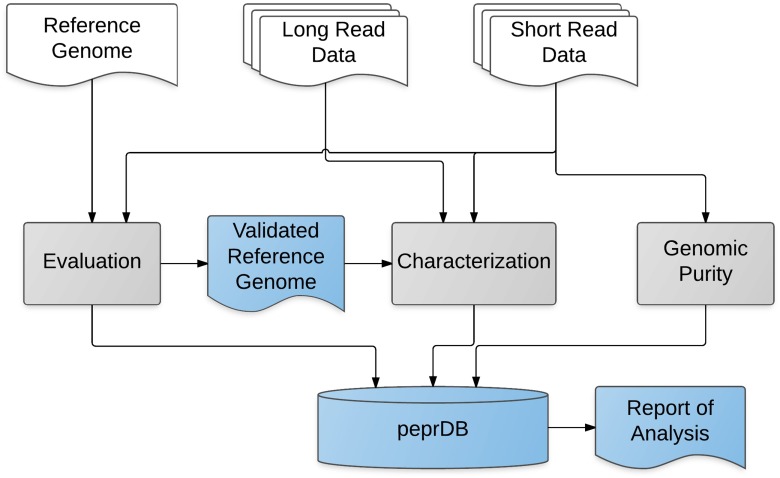
PEPR workflow. *White objects* are pipeline inputs, *grey objects* are the three pipeline components, and *light blue* objects are the pipeline products

PEPR consists of three bioinformatic pipelines written in Python (Fig. 1). The three bioinformatic pipelines are genome evaluation, genome characterization, and genomic purity. A YAML file (http://yaml.org) is used to define pipeline inputs. The pipeline coordinates the execution of a number of command line tools, logging the standard output and standard error for each executed command in time-stamped files for reference and debugging. Pipeline code is available at (https://github.com/usnistgov/pepr). To reduce the barrier for reuse, two Docker (https://www.docker.com/) containers are available with pre-installed pipeline dependencies. Docker is a lightweight virtual environment that facilitates the sharing and distribution of computing environments and can be run on any desktop, cloud, or high-performance computing environment, regardless of the operating system. The pepr container (https://registry.hub.docker.com/u/natedolson/pepr) includes dependencies for the genome evaluation and characterization pipelines, excluding the Genome Analysis Toolkit (due to licensing restrictions). The docker-pathoscope container has dependencies for the genomic purity pipeline installed (https://registry.hub.docker.com/u/natedolson/docker-pathoscope/).

A software package, peprr, was developed for the statistical computing language R [3] to compile the output from the genome evaluation, characterization, and genomic purity pipelines. The compiled data was formatted into a series of data tables within an SQLite, peprDB, database to facilitate downstream analysis [4]. The package includes functions to generate a number of summary tables and figures, including those in this publication.

### Genome evaluation pipeline

The Genome Evaluation Pipeline is the first step in the PEPR workflow and is used to reduce errors in the user-provided genome assembly prior to characterization. The evaluation pipeline consists of three steps. Illumina sequencing data are retrieved from the Genbank Sequence Read Archive (SRA) using the sratoolkit fastq-dump command (http://ncbi.github.io/sra-tools/). Users can also run the pipeline using fastq files by including file paths in the pipeline parameters file. Next, sequencing reads are mapped to the reference genome using BWA mem algorithm [5]. Finally, Pilon is used to evaluate and polish the reference assembly [6]. The corrected reference genome is then used as input for the Genome Characterization Pipeline.

### Genome characterization pipeline

The Genome Characterization Pipeline uses replicate sequence dataset from multiple sequencing platforms to characterize the corrected reference genome produced by the Genome Evaluation Pipeline at the individual base level. Illumina data are aligned to the reference genome using the same methods as the evaluation pipeline. Ion Torrent PGM data are mapped to the reference using the TMAP algorithm [7]. If Pacific Biosciences (PacBio) sequencing data are used to generate the input reference assembly, the data are mapped to the reference genome using the BWA mem algorithm [5]. Sequence alignment files are processed prior to downstream analysis by marking duplicates with Picard’s MarkDuplicates command (http://broadinstitute.github.io/picard) and realigning reads mapping to regions with insertions or deletion using the GenomeAnalysisToolKit [8, 9]. After refining the alignment files, base level analysis is performed using the short-read sequencing data. For each platform a VCF (variant call format) file with a number of summary statistics is generated using SAMtools mpileup [10]. A base purity metric is calculated from the resulting VCF files. The base purity metric is the number of high-quality bases (quality score ≥ 20) in reads aligned to a genome position that are in agreement with the reference base divided by the total number of reads high-quality bases supporting the reference and alternate base called by SAMtools. The metric is calculated from the SAMtools DP4 INFO tag in the vcf output generated with the mpileup command. Homogeneity analysis, a measure of genomic content similarity between vials of the reference material, is performed by first generating a pileup file using SAMtools mpileup for each dataset then performing pairwise tumor-normal variant calling using VarScan [11]. In this work, VarScan looks specifically for differences between vials in the proportion of reads containing variants. A standard Benjamini-Hochberg procedure was used to assess the power of the homogeneity analysis (Electronic Supplemental Material, https://github.com/DanSBS/NGSPower). Additionally, a number of summary statistics are calculated for the sequencing datasets using Picard’s Collect Multiple Metrics (http://broadinstitute.github.io/picard).

### Genome purity pipeline

The Genomic Purity Pipeline assesses the purity of the genomic material, defined as the presence of DNA from sources other than the expected genus. Material genomic purity was assessed using the metagenomic taxonomic read classification algorithm PathoScope 2.0 [12]. This method uses an expectation-maximization algorithm where the sequence data are first mapped to a database comprised of all sequence data in the Genbank nt database. Then, through an iterative process, PathoScope re-assigns ambiguously mapped reads to a taxonomic group based on the proportion of reads mapped unambiguously to individual taxonomic groups in the database. Using short-read sequencing data as input, PathoScope 2.0 first filters and trims low-quality reads (PathoQC), followed by mapping reads to a reference database (PathoMap - a wrapper for bowtie2 [13]), and then the expectation-maximization algorithm (PathoID) is used for the taxonomic classification. The annotated Genbank nt database provided by the PathoScope developers was used as the reference database (ftp://pathoscope.bumc.bu.edu/data/nt_ti.fa.gz).

### Candidate reference material *S. aureus* sequencing data

Sequencing data and the reference assembly for the NIST candidate reference material *S. aureus* was used to demonstrate how PEPR is used to characterize a genomic material. A *de novo* genome assembly from Pacific Biosciences (PacBio) long-read sequencing data was used as input for PEPR. Prior to being used as input, the assembly was validated using optical mapping data. Eight replicate vials of the candidate reference material were sequenced on the Illumina MiSeq and Ion Torrent PGM sequencing platforms (Electronic Supplemental Material).

## Results and discussion

Pipelines for Evaluating Prokaryotic References (PEPR) uses biological and technical replicate sequencing data from orthogonal sequencing platforms to characterize the reference genome of a prokaryotic material. The prokaryotic material is a batch of genomic DNA extracted from a prokaryotic culture. There are two primary reasons for using replicate sequencing datasets. One is to test for homogeneity within the batch of DNA. The second is to minimize the impact of library specific biases. The resulting characterized genome is suitable for evaluating and benchmarking whole genome sequencing methods. PEPR consists of three pipelines: genome evaluation, genome characterization, and genomic purity assessment (Fig. 1). The following section includes the characterization results for the NIST *S. aureus* candidate reference material along with a discussion of PEPR’s assumptions and limitations.

### Preparation of reference assembly

A user-provided high-quality closed reference assembly free of large assembly errors should be used as input when running PEPR. Optical mapping, as well as large insert mate-pair and synthetic long-read library preparation methods [14], are a few orthogonal methods that can be used to identify large mis-assemblies. The long DNA fragments used in optical mapping (average size < 200 Mb) allows for the identification of large mis-assemblies (< 3 kb) that are not easily identified using short-read sequencing data [15]. For the *S. aureus* RM, the reference assembly was constructed from Pacific Biosciences (PacBio) RSII long-read sequencing data (Electronic Supplemental Material). To validate the *S. aureus* reference assembly prior to evaluation with PEPR, OpGen optical mapping technology was used. The *S. aureus* optical mapping results were in agreement with the PacBio assembly, indicating no large mis-assemblies, and that the assembly was suitable for use in PEPR.

### Genome evaluation

The *Genome Evaluation* pipeline is the first step in PEPR. Pilon was chosen for the evaluation step as it assesses the accuracy of the genome and corrects errors in the assembly [6]. While currently not part of the PEPR, PAGIT, and REAPR are two alternative methods for evaluating and correcting genome assemblies similar to Pilon [16, 17]. Other methods are available for evaluating reference genomes, e.g. amosValidate [18] and ALE [19], however, these methods only assess assembly accuracy without correcting mis-assemblies. The resulting reference assembly represents the consensus genome of the population of cells used to generate the material. The genome evaluation pipeline does not attempt to identify or characterize low-frequency structural variants within the material or vial-to-vial variability of the reference genome. The evaluation pipeline failed to identify any assembly or base call errors in the *S. aureus* candidate genome assembly. The candidate genome, once evaluated, and, if necessary, refined during the *Genome Evaluation* pipeline, is used as input for the *Genome Characterization* pipeline.

### Genome characterization

The *Genome Characterization* pipeline calculates whole genome base level statistics using replicate sequencing data from orthogonal measurement methods. As part of the characterization pipeline, summary statistics are generated for the user provided sequencing datasets. While PEPR only uses short-read sequencing data to evaluate the material, dataset summary statistics for additional sequence data, such as long-read data used to generate the reference assembly, can be calculated. The results from our analysis of the *S. aureus* candidate reference material using PEPR provides an example of the type of information, summary figures, and tables that can be generated with PEPR.

#### Sequencing data summary statistics

Summary statistics were calculated including number of reads, mapped read length, insert size for paired-end datasets, and coverage for *S. aureus* datasets (Table 1). The MiSeq sequencing run had an average of 1.7 million paired-end reads per library with a median read length of 232 bp, whereas the PGM sequencing run produced 0.2 million reads per library on average with a median read length of 232 bp. Based on the sequencing methods used (Electronic Supplemental Material), longer reads were expected for PGM. The shorter read length is potentially due to the low GC content, which is known to challenge current sequencing technologies [20]. The higher throughput and paired-end reads resulted in higher coverage for MiSeq compared to PGM (251 X vs. 36 X). The three PacBio datasets are library replicates run on different SMRT cells. The replicate libraries had a median subread length 10,436 bp and 302 X total coverage. Between the three platforms a total coverage of 4,611 X. The dataset summary statistics provide general information about the sequencing datasets and identify potential biases in the sequence methods.

**Table 1:** Summary of sequencing datasets

Acc.	Plat	Vial	Lib.	Reads	Length (bp)	Insert (bp)	Cov.
SRR1979039	miseq	0	1	3305082	230	257	247
SRR1979040	miseq	0	2	3732088	216	233	263
SRR1979041	miseq	1	1	3973320	218	242	279
SRR1979042	miseq	1	2	3941040	223	247	285
SRR1979043	miseq	2	1	3442554	234	268	261
SRR1979070	miseq	2	2	3226726	232	268	240
SRR1979044	miseq	3	1	3025028	233	264	229
SRR1979045	miseq	3	2	4796382	200	210	303
SRR1979046	miseq	4	1	3338456	239	278	260
SRR1979047	miseq	4	2	2995090	237	277	231
SRR1979048	miseq	5	1	3495384	225	255	255
SRR1979049	miseq	5	2	3116128	241	281	244
SRR1979050	miseq	6	1	3129282	237	271	240
SRR1979060	miseq	6	2	2976312	242	280	233
SRR1979064	miseq	7	1	2630544	241	283	204
SRR1979065	miseq	7	2	3416580	225	248	247
SRR2002412	pgm	0	1	556903	231		42
SRR2002413	pgm	1	1	530117	224		38
SRR2002414	pgm	2	1	437527	231		33
SRR2002415	pgm	3	1	552692	232		42
SRR2002416	pgm	4	1	498479	232		37
SRR2002418	pgm	5	1	390070	235		30
SRR2002419	pgm	6	1	426196	232		32
SRR2002420	pgm	7	1	439119	238		34
SRR2056302	pacbio	9	1	163475	10510		108
SRR2056306	pacbio	9	2	163471	10436		103
SRR2056310	pacbio	9	3	163474	9863		91

Acc. - Sequence read archive (SRA) database accessions. Plat. - sequencing platform, miseq: Illumina MiSeq, pgm: Ion Torrent PGM, pacbio: Pacific Biosciences RSII. Lib. - library replicate number for miseq and pgm, smartcell replicate for pacbio. Reads - number of sequencing reads in the dataset. Length - median read length in base pairs. Insert - median insert size in base pairs for paired-end reads. Cov. - median sequence coverage across the genome

#### Base level purity

A base purity metric was used to evaluate how well the sequencing data supports the reference base call. Through comparison of the base purity for two orthogonal sequencing methods, we identified genome positions with low purity values due to platform specific systematic sequencing errors. The reference base is identified using a third orthogonal sequencing method (Pacific Biosciences RSII), which only chooses the dominant base and does not identify small impurities. Thus, a low purity (below 50 %) for one of the two short-read sequencing platforms and a high purity value for the other means that two technologies (one short-read and one long-read) agree that the dominant base is the reference base. It is important to acknowledge that even if the two short-read sequencing platforms indicate an impurity, they are potentially susceptible to the same unknown bias.

**Fig. 2:**
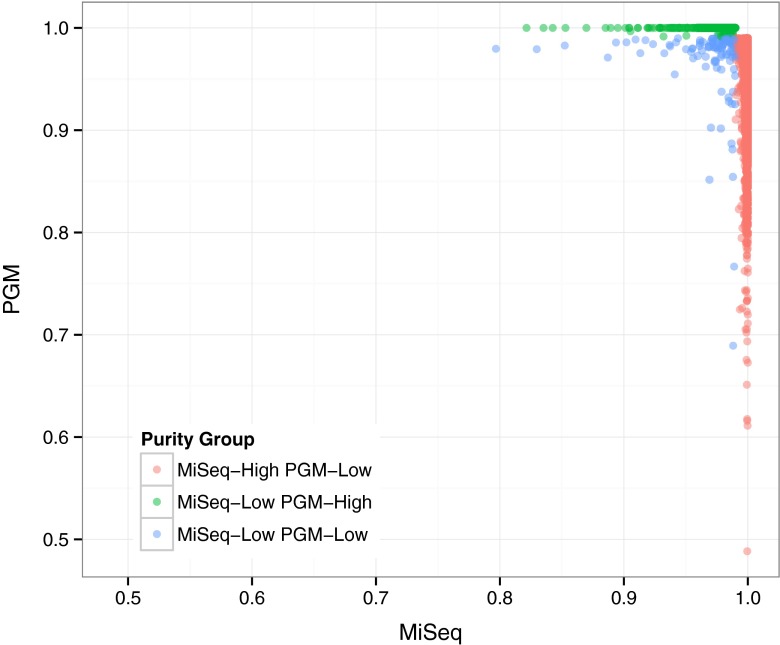
Comparison of base purity values for PGM and MiSeq. Positions are colored based of high and low purity values for the two sequencing platforms, MiSeq - Illumina MiSeq and PGM - Ion Torrent PGM. A purity value of 0.99 was used to differentiate between high and low purity positions. Positions with high purity for both platforms were excluded from the figure

**Fig. 3:**
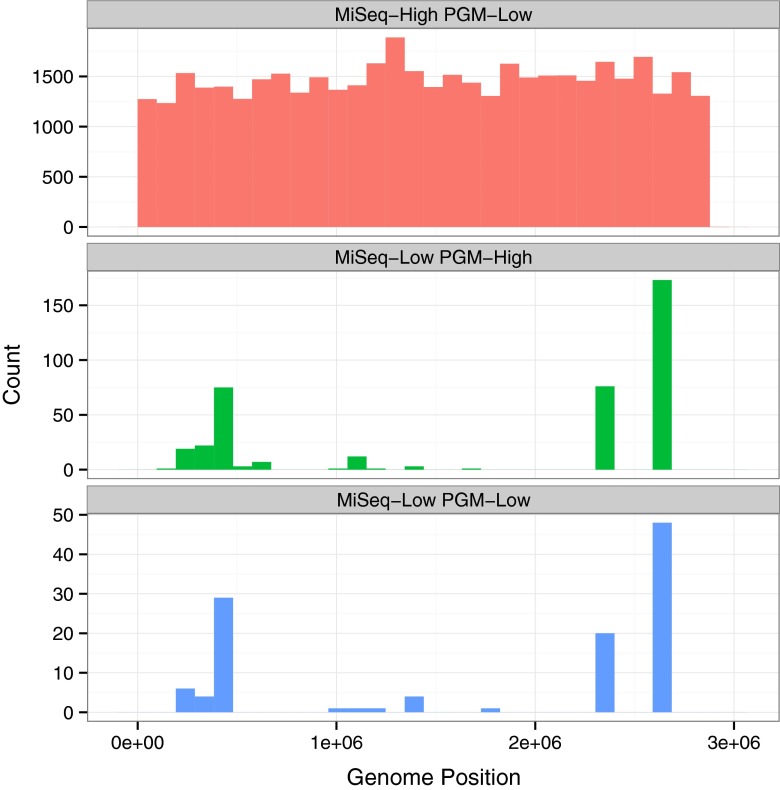
Distribution of genome positions by purity group. Bases with high and low purity and purity values greater than and less than 0.99 respectively for the two platforms, MiSeq - Illumina MiSeq and PGM - Ion Torrent PGM. Positions with high purity for both platforms were excluded from the figure

We compared purity metric values between two orthogonal sequencing methods, MiSeq and PGM, for all positions in the genome (Fig. 2). The purity metric was used to categorize genomic positions as high (> 0.99) or low purity (< 0.99). Out of 2,909,968 positions in the genome 2,864,925 positions had purity values greater than 0.99 for both short-read sequencing platforms (Table 2). Further, 2,909,853 and 2,909,965 positions had purity values greater than 0.99 and 0.97, respectively, for one of the two platforms. Only 115 positions had purity values less than 0.99 for both platforms, and no positions had purity value less than 0.95 for both platforms. The positions with low purity for MiSeq were non-uniformly distributed whereas positions with low purity for PGM were uniformly distributed (Fig. 3). The difference in low purity position distributions is due to differences in the systematic sequencing error profiles for the two platforms. PGM has a higher error rate for homopolymers, whereas MiSeq has a more context specific sequencing error profile [20].

**Table 2:** Number of genome positions with high and low purity, purity metric values higher and lower than 0.99 respectively, for the Illumina MiSeq and Ion Torrent PGM sequencing platforms

	PGM-High	PGM-Low
MiSeq-High	2864925	44534
MiSeq-Low	394	115

The sequencing technologies used to characterize the material are still maturing, and an incomplete understanding of platform-specific biases limits our ability to provide a confidence value for the base calls. A number of base level metrics, such as strand bias, are calculated as part of the PEPR Genome Characterization Pipeline and are included in the pipeline results database. These metrics can be used to differentiate positions with low purity due to measurement error and those due to biological variability. Use of additional metrics and algorithms developed for the identification of low-frequency variants, such as LoFreq [21], could help identify positions with low levels of biological variability, but are not currently implemented in PEPR.

#### Base level homogeneity

Material homogeneity was assessed through pairwise statistical analysis of the replicate MiSeq datasets using the VarScan somatic variant caller [11]. The pairwise variant analysis failed to identify any statistically significant base level differences among the replicates (Table 3). Only Illumina data was used to assess the homogeneity of the material as the higher coverage increased the statistical power of the test, and replicate libraries provide information regarding the method error rate. The Ion Torrent dataset did not include replicate libraries for the eight vials sequenced, and therefore, library specific sequencing errors were confounded with vial-to-vial variability. No statistically significant variants were identified between all pairwise comparisons indicating that the material is homogeneous. If potential inhomogeneities were found, then the PGM sequencing data could be examined for additional support for the inhomogeneities. Even without replicate libraries for the different vials, it is unlikely that any library specific bias will correlate with vial-to-vial variability observed in the Illumina data by chance.

**Table 3:** Pairwise variant analysis results

Position	Proportion of Pairs	Median Frequency	Minimum P-value	N Significant
244332	0.01	21.31	0.51	0.00
2615986	0.03	20.48	0.45	0.00
2616058	0.08	25.29	0.15	0.00
2619808	0.01	20.78	0.61	0.00
2619886	0.01	21.54	0.50	0.00

Position is the position in the genome where differences in variant frequency for at least one of the 16 pairwise comparisons were reported. Proportion of pairs is the fraction of the pairwise comparisons between the 16 Illumina MiSeq datasets where VarScan reported a difference in variant frequency. Median frequency is the median variant frequency for datasets with reported difference at that genome position. Minimum p-value is the lowest p-value reported by VarScan for all pairwise dataset comparisons with reported differences in variant frequency. N Significant is the number of datasets with reported statistically significant differences at that genome position

### Genomic purity pipeline

The *Genomic Purity* pipeline is used to identify DNA within the material that belongs to a genus other than the material genus. Short-read sequencing data was used to identify the proportion of DNA in the material from an organism other than the material genus, in this case, *Staphylococcus*, using PathoScope 2.0 [12]. The genus level cutoff was selected based on results from a previous study characterizing the specificity of the PathoScope 2.0 classification algorithm (Olson et al. *in-prep*). Genomic contaminants can be from the culture itself or reagents and materials used to prepare the material or during sequencing [22–24]. Contaminants identified by the Genomic Purity Pipeline may not be present in the material. For example, reagents used during library preparation may include contaminants [24–27]. Additionally, bioinformatic errors may lead to false positives, either due to errors in the database or errors by the classification algorithm.

**Fig. 4:**
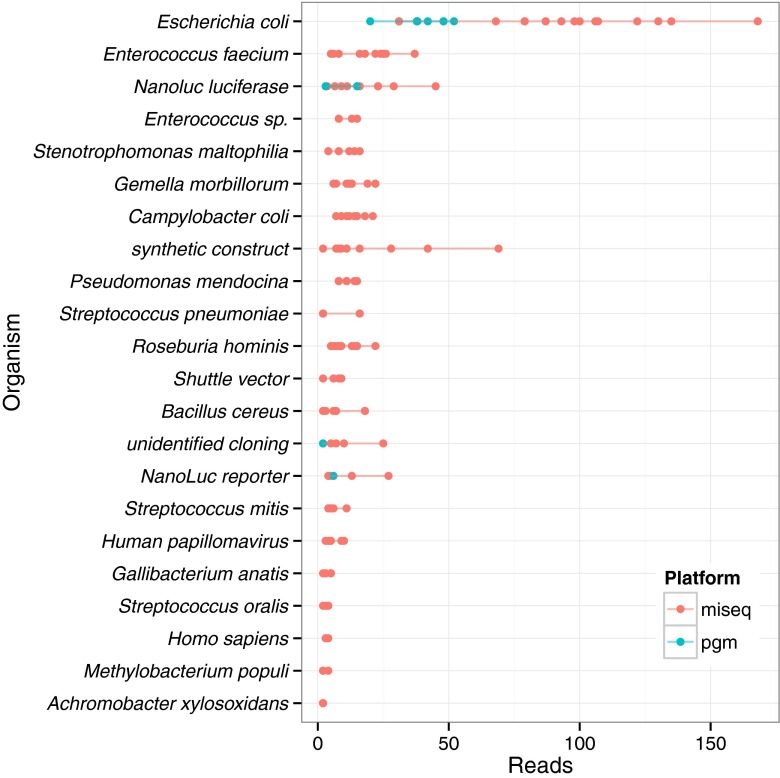
Breakdown of contaminants by organism

Based on analysis of the MiSeq and PGM sequencing data, the reference material has minimal if any genomic contaminants, with a maximum of 0.0039 % reads in any dataset classified as not belonging to the genus *Staphylococcus*. The most abundant contaminant was *Escherichia coli* (Fig. 4). *E. coli* is a well-documented contaminant of molecular biology reagents, and not likely a true contaminant [24]. Lower abundant contaminants may be bioinformatic errors and not true contaminants. While, contaminants identified by the *Genomic Purity* pipeline are most likely from reagents or due to bioinformatic errors, a conservative estimate of the material purity, assuming all contaminants are real, is reported by the pipeline. Users will want to consider the limited specificity of the taxonomic classification method. For example, if the intended use of the genomic DNA is for use as part of an inclusivity exclusivity panel, additional genomic purity assessment in addition to the PEPR Genomic Purity Pipeline is required to validate the material.

## Conclusions

PEPR provides a framework for characterizing microbial genomic reference materials, for instance, a homogenized batch of DNA from a single prokaryotic strain. The objective in developing PEPR was to provide a reproducible and transparent workflow for characterizing of prokaryotic genomic materials. The pipeline can be used to characterize reference materials as well as in-house quality control materials for which replicate sequencing datasets from multiple platforms are available. If another lab wishes to characterize a new reference or quality control material, they could follow this process:

1. Ideally, the user should generate a large batch of material and aliquot it to reduce inhomogeneity.
2. Identify a high-quality genome assembly. If a good reference assembly does not exist for the sample, then long-read sequencing like PacBio may be required to generate an assembly, and ideally mapping technologies would be used to validate the assembly.
3. Short-read whole genome sequencing, preferably from two orthogonal sequencing technologies, should be generated from multiple vials of the material, ideally with technical replicate libraries from at least six randomly selected vials.
4. PEPR can then be run to assess base level purity and homogeneity, genomic contaminants, and mis-assemblies.

PEPR outputs include a corrected reference genome assembly, genome positions with high and low purity based on biological and technical variation, base level homogeneity of the material, as well as the percentage and identity of genus level genomic contaminants. The resulting characterization values are intentionally conservative and without uncertainty or confidence estimates, as sources of bias and error associated with the measurement process are currently not fully understood. As the scientific community’s understanding of the measurement process matures new algorithms can be incorporated into the pipeline to increase the quality of material characterization process. The genomic materials characterized using PEPR will help increase confidence in WGS measurement methods and improve our understanding of the sequencing and data analysis process.

## Electronic supplementary material

Below is the link to the electronic supplementary material.

(PDF 1.02 MB)
