# Supplementary material for: PEPR: pipelines for evaluating prokaryotic references
Source: Anal Bioanal Chem. 2016 Mar 2;408:2975–83. doi: 10.1007/s00216-015-9299-5 (PMC4819933; doi:10.1007/s00216-015-9299-5)
Supplement: Supplementary file 1 — (PDF 1.02 MB) [file 216_2015_9299_MOESM1_ESM.pdf]

Analytical and Bioanalytical Chemistry

Electronic Supplementary Material

PEPR: pipelines for evaluating prokaryotic references

Nathan D. Olson, Justin M. Zook, Daniel V. Samarov, Scott A. Jackson, Marc L. Salit

Pipelines for Evaluating Prokaryotic References

Supplemental Material

Candidate Reference Material Sequencing and Genome Assembly

September 23, 2015

1 Material Development

Sequencing data for the NIST candidate reference material RM8376, genomic DNA from *Staphylococcus aureus* strain NRS100 isolate COL (Biosample SAMN02854573) was selected as a model system to demonstrate how PEPR is used to characterize a microbial genomic material. The strain was isolated from a clinical sample by Children's National Hospital (Biosample SAMN02700075). The genomic material represents a large homogeneous batch of extracted DNA, with 1500 vials with $3\mu g$ DNA in each. The extracted DNA was prepared from single colony of the initial culture stab was incubated overnight at 37 °C on an agar plate and a single colony was used to inoculate a new plate. One colony from the new plate was grown in 20 mL Luria-Bertani (LB) broth at 37 °C. The culture was used to inoculate 15 X 150 mm plates which were incubated at 37 °C for 16 hours. DNA was isolated by lysing the bacteria in a solution containing NaCl, Tris, EDTA and lysostaphin ($25\mu g/ml$) and SDS. Proteinase K and RNase A were used to treat protein and RNA. Ammonium acetate was used to remove protein. DNA was recovered by isopropanol precipitation as follows, the DNA was washed with 70% alcohol, drained, and dissolved in TE buffer (Tris 10 mM, EDTA 0.1 mM, pH8.0).

2 Sequencing Experimental Design

The *S. aureus* candidate reference materials were sequenced using three orthogonal sequencing platforms: Pacific Biosciences RSII (PacBio)[1], Ion Torrent PGM [2], and Illumina MiSeq [3]. For PacBio sequencing, the sequencing library was prepared using DNA Template Prep Kit 3.0 with pooled DNA from three randomly sampled vials of the candidate reference material RM8376. The resulting library was sequenced with the P6-C4 chemistry. For the Ion Torrent PGM [2] and Illumina MiSeq [3] sequencing, eight

[1]Pacific Biosciences of California Inc.`http://www.pacificbiosciences.com/` 1380 Willow Rd. Menlo Park, CA 94025 USA

[2]Life Technologies Corp., `http://www.iontorrent.com/` 7000 Shoreline Court # 201, South San Francisco, CA 94080 USA

[3]Illumina Inc., `http://www.illumina.com/` 5200 Illumina Way San Diego, CA 92122 USA

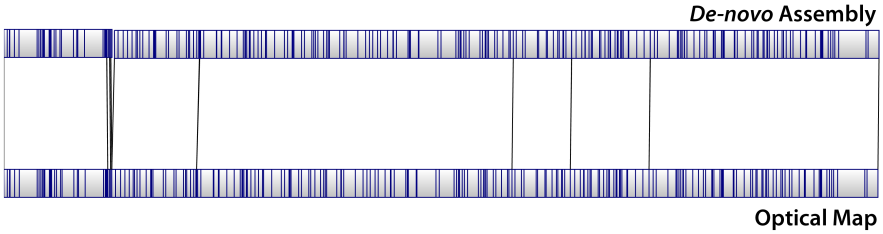

Figure 1: Comparison of optical map data to genome assembly. Alignment of *in-silico* genome map generated from the PacBio HGAP assembly to the OpGen optical map. Blue bars in map represent NocI restriction sites, black lines indicate co-linear regions.

vials were randomly sampled from the lot of 1500 vials. For MiSeq, two technical replicate libraries were prepared for each of the eight vials using the Nextera DNA Sample Prep Kit[3]; samples were barcoded using the Nextera Index Kit and sequenced using the MiSeq 600 cycle Reagent kit v3 for 2 X 300 bp reads. The 16 libraries were pooled and sequenced in a single run. Single barcoded 400 bp Ion Torrent PGM libraries were prepared for each of the eight vials using the Ion Xpress Plus kit [2]. The vials were barcoded using the IonXpress Kit[3], sequencing template was prepared using the Ion PGM Template OT2 400 kit and the IonPGM400 kit was used for sequencing on a 318C chip. The raw sequence data is archived in the Genbank Sequence Read Archive (http://www.ncbi.nlm.nih.gov/sra), see Table 1 of main paper for accession numbers. A genome map was obtained for the candidate reference material from optical mapping data generated using the Argus Optical Mapping System[4]. Agarose plugs generated from the same culture stock as that used to generate the DNA reference material were used for optical mapping measurement. Restriction enzyme NcoI was used for the restriction digest. These steps occured prior to our PEPR pipeline.

3 *S. aureus* Reference Assembly

PacBio long read data were used to generate a *de novo* genome assembly for use in the Genome Evaluation Pipeline. PacBio Reads were assembled using SMRTAnalysis software version 2.3 [1] to apply the HGAP assembly algorithm [1]. To identify potential errors in the assembly, an *in silico* digest of assembly was compared the genome map obtained from the optical mapping data using OpGen MapSolver software[4]. The *de novo* assembly was supported by Optical mapping data (Fig. 1). The plasmid is too small for OpGen optical mapping technology, and therefore the plasmid assembly was not validated with the optical mapping data.

[4]OpGen Inc. `http://opgen.com` 708 Quince Orchard Road Gaithersburg, MD 20878 USA

References

[1] Sergey Koren et al. "Reducing assembly complexity of microbial genomes with single-molecule sequencing." In: *Genome biology* 14.9 (2013), R101. ISSN: 1465-6914. DOI: 10.1186/gb-2013-14-9-r101. arXiv: 1304.3752. URL: http://www.pubmedcentral.nih.gov/articlerender.fcgi?artid=4053942\&tool=pmcentrez\&rendertype=abstract.

Sequencing Error Adjusted and Multiple Comparison Corrected Read Depth Estimation

Introduction

A critical component in planning Next Generation Sequencing (NGS) experiments is determining appropriate read depth. This is particularly important for reliably detecting what if any differences exist between two biological samples, for example normal versus tumor tissue. The problem of determining appropriate read depth can be thought of as a sample size estimation problem with two important considerations. The first is the inclusion of systematic sequencing errors (SSE) on read accuracy; this can include quality score and other metrics. The second is controlling for multiple comparisons, which in the context of NGS is especially important as we are comparing millions/billions of locations between the two samples.

In this analysis we develop a Bayesian method for helping to determine read depth and associated power estimates, taking into account both sequencing error and false discovery rate. Our specific focus will be to provide read depth estimates in the context of sample homogeneity assessment; i.e. looking at two sequences grown from the same batch and determining how deeply those sequences need to be sequenced in order to detect whether any differences exist.

Sampling Distribution

As this type of analysis is done prospectively to help determine appropriate read depth coverage for the final sample preparations, we work with simulated data only as no measurements have been taken. In order to arrive at an estimate of read depth we need a way to simulate *observed* purities, i.e. adjusted for SSEs and other distributional factors associated with an NGS experiment. Our objective is to compare the purities, simulated from two vials and determine if differences could be detected as we slowly change the underlying *true* purity of one vial, while holding the other fixed. Stated in terms of a hypothesis test we have

$$H_0\text{: purity of vial}_1 = \text{purity of vial}_2 \text{ vs. } H_A\text{: vial purities not equal.}$$

To generate these purities we used a Bayesian sampling scheme as a first step in our analysis, this is done prior to any adjustment for multiple comparisons which we describe in the following section. We now describe the sampling procedure.

We begin by defining the terms of our model (note that these all pertain to the sampling of a purity associated with a base at a given position in the genome for a single vial), letting denote r read depth we have

$B_i =$ The event that the reference base $b \in \{A, C, T, G\}$ is matched in read $i, i = 1, \ldots, r$,
$E_i =$ The occurence of a sequencing error at read $i, i = 1, \ldots, r$,

For each of these we assume the following probability distributions:

$$(B_i|E_i = 0, p_b) \sim Bernoulli(p_b), i = 1, \dots, r$$
$$(B_i|E_i = 1, p_b) \sim Bernoulli(1 - p_b), i = 1, \dots, r$$
$$E_i \sim Bernoulli(\epsilon), i = 1, \dots, r$$
$$\Pr(p_b) = Beta(\alpha, \beta)$$

The conditional distributions of the data can be understood as follows. For the case given by $(B_i|E_i = 0, p_b)$, i.e. where no sequencing errors have occurred, the probability of observing the correct reference base is related to the purity of that base, p_b and follows a $Bernoulli(p_b)$ distribution. For the case where a sequencing error *has* occurred, $(B_i|E_i = 1, p_b)$ the conditional distribution captures the event that the incorrect base is sampled but is identified as the reference base. For this reason the appropriate distribution for capturing this event is $Bernoulli(1 - p_b)$. An illustration of our model is shown in Figure 1 below.

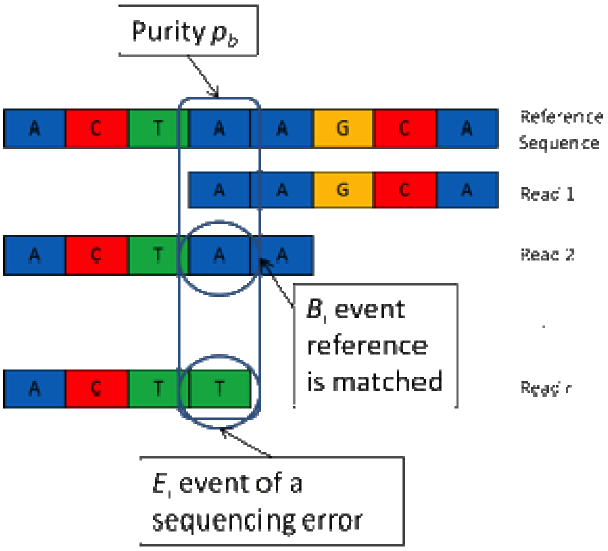

Fig. 1 Model for purity of a base and sequencing errors

Of primary interest is to compute the posterior of p_b given the observed data B_i conditional on the error parameter E_i, which can be shown to be

$$p_{obs} \sim \Pr(p_b|B_i, i = 1, \dots, r)$$
$$\propto \prod_{i=1}^{r} [p_b^{B_i}(1 - p_b)^{1-B_i}(1 - \epsilon) + p_b^{1-B_i}(1 - p_b)^{B_i}\epsilon] \times p_b^{\alpha-1}(1 - p_b)^{\beta-1}.$$

In order to sample p_{obs} we must first generate our observed data B_i. To do this we begin by sampling a set of r sequencing errors E_i with a fixed error rate ϵ (though it should be noted that these errors could be allowed to vary at each position based on quality scores, etc.). With these we then sample our observed data as

$$B_i \sim \frac{B(\alpha+k,\beta-k+1)}{B(\alpha,\beta)} = Bernoulli\left(\frac{\alpha}{\alpha+\beta}\right), \text{if } E_i = 0 \text{ and}$$
$$B_i \sim \frac{B(\alpha-k+1,\beta+k)}{B(\alpha,\beta)} = Bernoulli\left(\frac{\beta}{\alpha+\beta}\right), \text{if } E_i = 1.$$

Here $B(\alpha,\beta)$ is the beta function which is defined as

$$B(\alpha,\beta) = \frac{\Gamma(\alpha)\Gamma(\beta)}{\Gamma(\alpha+\beta)},$$

with $\Gamma(z) = \int_0^\infty t^{z-1}e^{-t}dt$ being the gamma function. With the observed samples B_i we can now use Markov Chain Monte Carlo (MCMC) to sample p_{obs}. For our analysis MCMC was implemented using the `rjags` package in the R programming language.

Generating null and alternative distributions

With a way to sample the observed purities p_{obs}, our objective is to estimate the probability of detecting differences in purities between vials as we vary the *true* purity p_b (i.e. the *power* of the test).

To do this we begin by estimating the null distribution, i.e. when the distribution of p_b is the same for both vials 1 and 2, specifically $\alpha_1 = \alpha_2$ and $\beta_1 = \beta_2$, here the subscripts denote the corresponding vials parameters for the Beta distribution (note, this sampling is for a given read depth r). Recall that the expected value of the beta distribution is $\frac{\alpha}{\alpha+\beta}$, so that the ratio of these parameters defines the underlying pure probability. In our analysis we consider two scenarios

1. Vial 1 purity p_b with expectation $\frac{\alpha_1}{\alpha_1+\beta_1} = 0.99$, with vial 2 expectation $\frac{\alpha_2}{\alpha_2+\beta_2} \in \{0.98, 0.96, \ldots, 1\}$.
2. Vial 1 purity p_b with expectation $\frac{\alpha_1}{\alpha_1+\beta_1} = 0.50$, with vial 2 expectation $\frac{\alpha_2}{\alpha_2+\beta_2} \in \{0.48, 0.46, \ldots, 1\}$.

These are meant to capture the two cases of observed purities we would expect to see in experimental settings.

Given scenarios 1 and 2, let $p^0_{obs,j}(1)$ and $p^0_{obs,j}(2)$, $j = 1, \ldots, J$ denote draws from the null distribution for vials 1 and 2 respectively, define $d^0_j = p^0_{obs,j}(1) - p^0_{obs,j}(2)$ to be the difference in the observed purities and $d^0 = (d^0_1, \ldots, d^0_J)$ the collection of these differences. In a similar fashion we make draws from the alternative distribution, altering the expectation for vial 2 as defined above; for this case define $d^1 = (d^1_1, \ldots, d^1_J)$ as the collection of these differences. With d^0 and d^1 we can now estimate the distribution of the differences for the null and alternative hypotheses. To do this we use the binned kernel density estimate function, `bkde` in R.

Computing the power of the test

Pictorially the power of our test, i.e. the probability of detecting a difference when one exists is shown below in Figure 2. Here the starting point of the green region (the power) is determined by the pre-determined level of significance γ, i.e. the probability of rejecting the null when no differences exist. Typically this value is taken to be $\gamma = 0.05$, but as will be discussed in the

following section, in order to account for multiple comparisons we use an adjustment of this to determine the starting point for our power estimate.
Irrespective the cutoff associated with a particular value of γ is established by finding the quantile from the binned kernel density fit to the sampled differences in d^0. The area under the alternative distribution for d^1 is then computed and corresponds to our estimate of power.

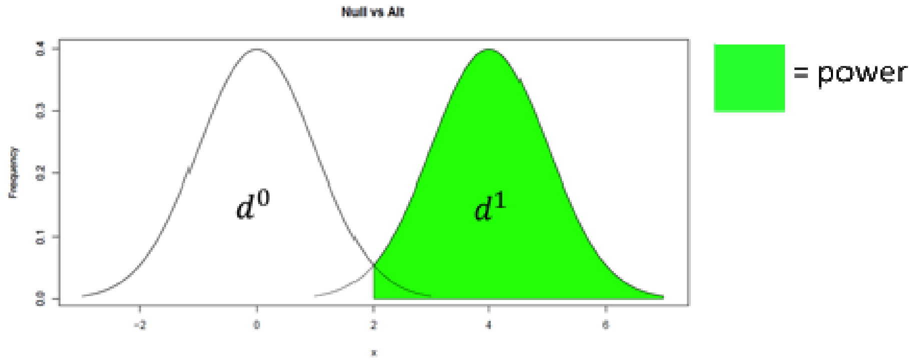

Fig. 2 Pictorial description of the power to detect significant difference between two distributions

Adjusting for multiple comparisons

In order to adjust for multiple comparisons we use the well-known Benjamini-Hochberg (BH) adjustment for controlling False Discovery Rate (FDR). FDR procedures are designed to control the expected proportion of incorrectly rejected null hypotheses, i.e. false discoveries. In contrast, multiple comparison correction using family wise error rate (FWER) based procedures, such as the Bonferroni correction, seek to reduce the probability of even one false discovery, as opposed to expected proportion of false discoveries. Thus FDR procedures have great *power* at the cost of increased rate false positives.

In its most common form the BH adjustment calls for taking the computed p-values and ordering, smallest to largest as $P_{(1)} \leq P_{(2)} \leq \cdots \leq P_{(G)}$, where G denotes the number of positions in the genome we are computing pairwise comparisons for. The procedure then proceeds by finding the largest k such that $P_{(k)} \leq \frac{k}{G}\gamma$. Intuitively we can think of the proportion $\frac{k}{G}$ as representing the number of pairwise differences we might expect to see *correctly* rejected. Put another way, if some number of difference are observed between vials at various positions in the genome, this proportion informs how many of those differences we expect to be *real* versus artifacts of the large number of comparisons or feature of the experimental process. So, for example, taking $\frac{k}{G} = 1.0$ would basically say that we believe all detected differences are real. The impact that this adjustment has on our power estimate is shown in Figure 3.

As the number of actual differences between sequences should be small so to should the ratio $\frac{k}{G}$, however, certain "real" differences do occur so that $k > 0$. So, while many differences may be detected, largely as a result of the massive number of comparisons being made, we believe only a small number of these reflect actual differences. For our purposes we

conservatively set $\frac{k}{G} = 0.05$, i.e. of the differences that are detected, we believe that only 5% may be attributable to real events (and many of these may be related to sequencing artifacts).

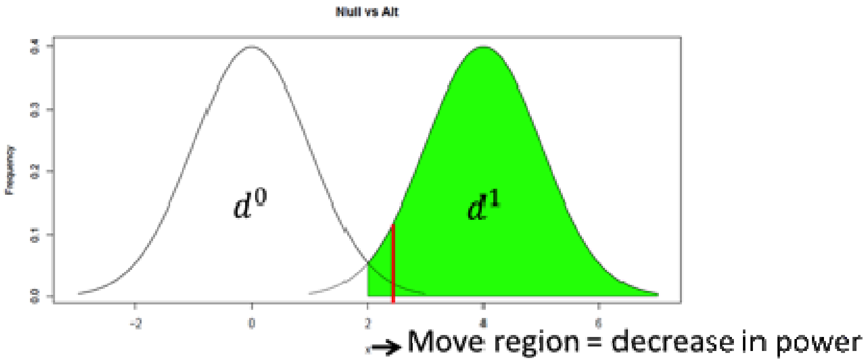

Fig. 3 Pictorial depiction of decrease in power to detect differences due to multiple comparisons
